# Supplementary material for: Effect of Changes in Veterinary Feed Directive Regulations on Violative Antibiotic Residues in the Tissue of Food Animals from the Inspector-Generated Sampling in the United States
Source: Microorganisms. 2022 Oct 14;10(10):2031. doi: 10.3390/microorganisms10102031 (PMC9612137; doi:10.3390/microorganisms10102031)
Supplement: Supplementary file 1 [file microorganisms-10-02031-s001.zip › microorganisms-1815511-supplementary.pdf]

Supplementary Materials:

Table S1. Univariable association between predictors and detection of violative residues of Desfuroylceftiofur in the tissue of food animals (*n*=809) from the IGS, 2014-2019.

Predictor	Categories	Violation N (%)	Non-violation N (%)	OR	95% CI	*P*-value
VFD rule change						0.765
	Before VFD rule change (2014-2016)	522 (87)	81 (13)	Referent		
	After VFD rule change (2017-2019)	180 (87)	26 (13)	1.07	0.66, 1.72	0.767
Animal production class						0.963
	Bob veal	37 (88)	5 (12)	1.14	0.43, 3.03	0.782
	Beef cow	42 (88)	6 (12)	1.08	0.44, 2.66	0.858
	Dairy cow	387 (87)	60 (13)	Referent		
	Bull	6 (100)	0 (0)	1	NA	NA
	Heifer	203 (88)	28 (12)	1.12	0.69, 1.81	0.633
	Steer	18 (82)	4 (18)	0.69	0.22, 2.13	0.528
	Goat	9 (82)	2 (18)	0.69	0.14, 3.30	0.650
	Sheep	0 (0)	1 (100)	1	NA	NA
	Swine	0 (0)	1 (100)	1	NA	NA
	Turkey	NA	NA	NA	NA	NA
Type of tissue sampled						0.001
	Others (muscle/liver)	4 (36)	7 (64)	Referent		
	Kidney	698 (87)	100 (13)	12.21	3.51, 42.47	<0.001

95% confidence interval (CI); odds ratio (OR); NA (not applicable).

Table S2. Univariable association between predictors and detection of violative residues of tilmicosin in the tissue of food animals (*n*=207) from the IGS, 2014-2019.

Predictor	Categories	Violation N (%)	Non-violation N (%)	OR	95% CI	*P*-value
VFD rule change						0.739
	Before VFD rule change (2014-2016)	74 (69)	33 (31)	Referent		
	After VFD rule change (2017-2019)	67 (67)	33 (33)	0.90	0.50, 1.62	0.739
Animal production class						0.855
	Bob veal	43 (81)	10 (19)	1.43	0.49, 4.11	0.504
	Beef cow	28 (78)	8 (22)	1.16	0.38, 3.58	0.788
	Dairy cow	24 (75)	8 (25)	Referent		
	Bull	12 (75)	4 (25)	1	0.25, 3.99	1.000

	Heifer	20 (77)	6 (23)	1.11	0.33, 3.73	0.865
	Steer	11 (65)	6 (35)	0.611	0.17, 2.19	0.450
	Goat	3 (60)	2 (40)	0.49	0.07, 3.54	0.488
	Sheep	NA	NA	NA	NA	NA
	Swine	0 (0)	12 (100)	1	NA	NA
	Turkey	0 (0)	10 (100)	1	NA	NA
Type of tissue sampled						
	Others (muscle/liver)	141 (76)	44 (24)	Referent		
	Kidney	0 (0)	22 (100)	1	NA	NA

95% confidence interval (CI); odds ratio (OR); NA (not applicable).

Table S3. Univariable association between predictors and detection of violative residues of florfenicol in the tissue of food animals (*n*=181) from the IGS, 2014-2019.

**Predictor**	**Categories**	**Violation N (%)**	**Non-violation N (%)**	**OR**	**95% CI**	***P*-value**
VFD rule change						0.3306
	Before VFD rule change (2014-2016)	70 (76)	22 (24)	Referent		
	After VFD rule change (2017-2019)	62 (70)	27 (30)	0.72	0.37, 1.39	0.332
Animal production class						0.1737
	Bob veal	42 (78)	12 (22)	2.39	0.92, 6.21	0.073
	Beef cow	22 (71)	9 (29)	1.67	0.58, 4.77	0.336
	Dairy cow	19 (59)	13 (41)	Referent		
	Bull	14 (93)	1 (7)	9.57	1.11, 82.06	0.039
	Heifer	13 (62)	8 (38)	1.11	0.35, 3.43	0.854
	Steer	17 (77)	5 (23)	2.32	0.68, 7.89	0.175
	Goat	3 (75)	1 (25)	2.05	0.19, 21.97	0.552
	Sheep	2 (100)	0 (25)	1	NA	NA
	Swine	NA	NA	NA	NA	NA
	Turkey	NA	NA	NA	NA	NA
Type of tissue sampled						
	Others (muscle/liver)	124 (72)	49 (28)	Referent		
	Kidney	8 (100)	0 (0)	1	NA	NA

95% confidence interval (CI); odds ratio (OR); NA (not applicable).

Table S4. Results of multivariable logistic regression for predictors of detection of violative residues of Desfuroylceftiofur in the tissue of food animals (*n*=809) from the IGS, 2014-2019.

**Predictor**	**Categories**	**OR**	**95% CI**	***p*-value**
VFD rule change				0.963
	Before VFD rule change (2014-2016)	Referent		
	After VFD rule change (2017-2019)	0.98	0.61, 1.59	0.964
Type of tissue sampled				0.001
	Others (muscle)	Referent		
	Kidney	12.24	3.50, 42.85	<0.001

95% confidence interval (CI); odds ratio (OR).

Table S5. Results of multivariable logistic regression for predictors of detection of violative residues of tilmicosin in the tissue of food animals (*n*=207) from the IGS, 2014-2019.

**Predictor**	**Categories**	**OR**	**95% CI**	***p*-value**
VFD rule change				0.739
	Before VFD rule change (2014-2016)	Referent		
	After VFD rule change (2017-2019)	0.90	0.50, 1.62	0.739

95% confidence interval (CI); odds ratio (OR).

Table S6. Results of multivariable logistic regression for predictors of detection of violative residues of florfenicol in the tissue of food animals (*n*=181) from the IGS, 2014-2019.

**Predictor**	**Categories**	**OR**	**95% CI**	***p*-value**
VFD rule change				0.330
	Before VFD rule change (2014-2016)	Referent		
	After VFD rule change (2017-2019)	0.72	0.37, 1.39	0.332

95% confidence interval (CI); odds ratio (OR).
